# From Upper Respiratory Symptoms to Hemophagocytic Lymphohistiocytosis: Case Report of a Human Adenovirus Infection in Haploidentical Hematopoietic Stem Cell Transplant Recipient

**DOI:** 10.3390/pathogens10030340

**Published:** 2021-03-15

**Authors:** Baptiste Demey, Clément Brault, Julien Maizel, Catherine Francois

**Affiliations:** 1 Department of Virology, Amiens University Medical Center, 80000 Amiens, France; catherine.francois@u-picardie.fr; 2 AGIR Research Unit, EA 4294, Jules Verne University of Picardie, 80000 Amiens, France; 3 Intensive Care Department, Amiens University Medical Center, 80000 Amiens, France; brault.clement@chu-amiens.fr (C.B.); maizel.julien@chu-amiens.fr (J.M.)

**Keywords:** adenovirus, hematopoietic stem cell transplant, lymphohistiocytosis, respiratory infection

## Abstract

Human adenovirus infection is rare in adult population, except for in immunocompromised individuals. Recipients of allogenic haploidentical hematopoietic stem cell transplantation are reported at high risk for human adenovirus, which is often lethal when it evolves into the disseminated form. Despite existent guidelines, prevention, early diagnosis, and therapeutics remain challenging. Here, we report the case of a fatal evolution of human adenovirus respiratory infection and discuss the actual recommendations to prevent recurrence of this major issue.

## 1. Introduction

Human adenovirus (HAdV) is a rare infection in adult population but can be fatal for immunocompromised patients. Recipients of allogenic hematopoietic stem cell transplantation (HSCT), especially haploidentical (haplo-) HSCT, present higher risk of HAdV reactivation or primary infection, due to immunosuppressive regimens. HAdV infection mostly occurs within the 100 days post-transplant [1] and could be asymptomatic, localized (e.g., respiratory disease, intestinal manifestations, or hemorrhagic cystitis) or could evolve to a disseminated HAdV disease (characterized by HAdV DNA-emia). Disseminated HAdV disease leads to a 20–80% rate of death [2], and Taniguchi et al. showed that up to 5.8% of haplo-HSCT recipients develop this lethal form of infection [3]. Recommendations concerning HAdV monitoring for high-risk patients were issued in 2012 by the European Conference on Infections in Leukaemia (ECIL)-4 group [4], with an update by the ECIL-8 group for community-acquired respiratory virus [5]. Nevertheless, the guidelines remain insufficient to establish an early and efficient diagnosis of this frequently fatal infection. Nowadays, syndromic approaches and the expansion of molecular analyses offer us new screening tools to prevent disseminated HAdV infections and define new recommendations.

## 2. Case Presentation

**Figure 1:**
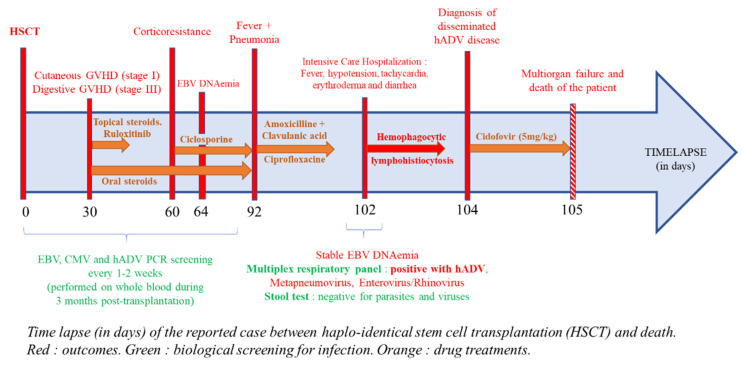
Time lapse (in days) of the reported case between haplo-identical stem cell transplantation (HSCT) and death. Red: outcomes. Green: biological screening for infection. Orange: drug treatments.

A 31-year-old man received a haplo-HSCT with sequential conditioning to treat a diffuse large B-cell lymphoma. Outcomes, drug treatments, and biological screening for infections after the HSCT are reported in Figure 1. After 30 days post-transplant, we observed a complete (100%) donor chimerism. He developed an acute cutaneous graft versus host disease (GVHD) (stage I, grade I) treated with topical corticosteroids. Due to the occurrence of a severe (stage III) digestive GVHD, a high dose of corticosteroids (2 mg/kg) and ruloxitinib (10 mg twice a day) were started. Ruloxitinib was rapidly stopped due to thrombopenia. Due to corticosteroids resistance, ciclosporin was introduced two months post-transplant and allowed complete remission of cutaneous and digestive GVHD. Maintenance corticosteroids (0.5 mg/kg per day) were continued. During the 3 months post-transplant, the patient was screened with polymerase chain reaction (PCR) in peripheral blood (PB) every 1–2 weeks for cytomegalovirus (CMV), Epstein–Barr virus (EBV), and HAdV as recommended. All CMV and HAdV PCRs remained negative. Recurrent EBV DNA-emia was observed and treated by two rituximab administrations, leading to a significant decrease in viral load. Among further post-allograft infections, we noticed genital herpes and a *Bacteroides uniformis* bacteremia, which were treated by acyclovir and amoxicillin/clavulanic acid, respectively.

Three months after haplo-HSCT, the patient consulted his general practitioner for fever, persistent coughing, and greenish sputum suggesting pneumonia. Probabilistic antibiotic by amoxicillin/clavulanic acid and ciprofloxacin was started for 7 days. Both blood culture and cytobacteriological examination of sputum were negative. A whole-blood EBV PCR was performed and showed a stable viral load. Ten days later, because his condition rapidly deteriorated, he was admitted in the Intensive Care Department. He presented a fever up to 40 °C (104 °F), arterial hypotension (90/50 mmHg), and tachycardia (140 beats per minute). Clinical examination showed erythroderma and diarrhea. Laboratory findings showed an important liver cytolysis (alanine aminotransferase superior to 1000 IU/L), triglycerides greater than 4 g/L, and ferritin level over 200,000 microg/L. The H-Score was 257, corresponding to a probability of hemophagocytic lymphohistiocytosis (HLH) of 99.6%. In the context of pneumonia, multiplex PCR for respiratory viral panel (FilmArray RP2 plus, BioMerieux, Marcy l’Etoile, France) was performed on nasopharyngeal swab. This test highlighted the presence of HAdV, metapneumovirus, and enterovirus/rhinovirus (indistinguishable). Stool analyses were negative for parasites, rotavirus, norovirus, and HAdV F40/41. PB PCRs were negative for *Toxoplasma gondii* and CMV. However, EBV and HAdV were detected with a 2.71 and 9.44 log copies/mL viral load, respectively. Cidofovir (5 mg/kg) was immediately introduced. Unfortunately, the patient died the next day due to a septic shock with multiorgan failure.

## 3. Discussion

We were faced with a rare clinical evolution of an HAdV infection, marked by a hemophagocytic lymphohistiocytosis. This association had already been described in pediatric population after HSCT [6,7], but rarely in adult population [8,9]. This is the first case described in a haplo-HSCT recipient while the practice of routine PCR monitoring and multiplex assays based on syndromic approaches spreads in laboratories.

HAdV infection is not common in adult population, but the risk is highly increased after haplo-HSCT. Other risk factors of HAdV infection presented by our patient are GVHD and a higher need for immunosuppressive agents [10]. However, the actual recommendations are not precise enough to cover patients with such risk factors. In fact, ECIL-4 recommendations indicate that HAdV screening should be considered in high-risk allogenic HSCT patients, such as those receiving haploidentical and umbilical cord blood transplantation. Quantitative PCR (qPCR) is the gold standard for the screening and diagnosis of HAdV infection. Molecular biology helps to identify patients at high risk when performed in stool samples. Still, according to ECIL-4 recommendations, length of screening should be adapted in accordance with immune reconstitution. Although these elements should be useful, the information can be interpreted and applied in different ways. During ECIL-8, experts indicated that nucleic acid testing (NAT) detecting community-acquired respiratory virus, such as HAdV, genomes is the preferred method to confirm the respiratory tract infectious disease [5]. We do not have guidelines in cases of HAdV infection without respiratory symptoms.

In the present case, and as is usual in our center, HAdV was screened in whole blood using qPCR during the first 3 months following HSCT. However, it only highlights HAdV DNA-emia, marker of systemic infection, which is necessary to start a treatment but appears at a stage of infection that could reach a 53% rate of mortality in immunocompromised hosts [11]. Lion et al. suggested an algorithm for diagnosis and treatment of HAdV infections based on stool analysis to obtain an earlier diagnosis [12]. The AdVance study reported 55% of allogenic-HSCT recipients who developed HAdV viremia showed preliminary HAdV detection on stool, urine, or respiratory secretions. Taniguchi et al. noticed that disseminated HAdV infection was always associated with hemorrhagic cystitis [3]. The most recent version of FilmArray respiratory panel (RP2plus) presents high sensibility and specificity for HAdV (respectively 94.6% and 96.9%) [13]. According to those observations, in the absence of supplemental data, a study evaluating the relevance of peripheral screening on different sample types (stool, urine, and respiratory secretions) is necessary. Moreover, an updated clear and consensual HAdV screening algorithm, based on systemic and organ-specific symptoms, matching with clinical practice, should be defined to make the prevention and diagnosis of HAdV diseases easier. During ongoing systematic monitoring, HAdV should be searched for, specifically or with syndromic approach, in hosts at high risk when they present symptoms compatible with HAdV infection. In the present case, HAdV could have been detected in different samples on the basis on nonspecific symptoms for this haplo-HSCT recipient case. Because of his increased risk of HAdV infection, the virus could have been searched through FilmArray RP2plus in the presence of signs of pneumopathy. Fever should motivate HAdV quantitative PCR in peripheral blood, and in the present case, this analysis could have justified an earlier start of treatment (10 days before). As for the invasive fungus infection, when fever persists despite broad-spectrum antibiotic therapy and without bacteria documentation, systemic viral infection would be suspected. When gastric symptoms appeared, a quantitative PCR targeting all types of HAdV should have been preferred to an antigenic rapid diagnostic test that detect restricted HAdV subtypes in stools (i.e., F40 and F41 only). Medical staff observed hematuria during hospitalization, but etiology of hemorrhagic cystitis was not explored. Frequency of HAdV urinary disease during disseminated infection [14] justified a quantitative PCR in urine. Obviously, investigations for HAdV would be complementary to analyses looking for bacteria, fungi, and other viral agents (such as CMV, EBV, HHV6, and BK polyomavirus). Earlier diagnosis of HAdV infection is essential to maximize patient survival and initiate cidofovir therapy, in default of more specific and effective treatment.

## 4. Conclusions

Here, we reported a case of a haplo-HSCT recipient who developed a respiratory HAdV infection that led to a hemophagocytic lymphohistiocytosis and the death of the patient. With adapted HAdV screening based on updated consensual recommendations and syndromic PCR tools, this type of infection could have been diagnosed earlier and such outcomes prevented. HAdV infection must be explored if respiratory, intestinalis, urinary, or systemic symptoms occur in immunocompromised patients, especially in haplo-HSCT recipients.

## Data Availability

The data presented in this study are available on request from the corresponding author. The data are not publicly available due to privacy and ethical restrictions.
